# Pulmonary Benign Metastasizing Leiomyoma in a Postmenopausal Woman: A Case Report and Review of the Literature

**DOI:** 10.3390/diseases12080181

**Published:** 2024-08-11

**Authors:** Aleksandra Piórek, Adam Płużański, Piotr Wiśniewski, Sylwia Tabor, Kinga Winiarczyk, Magdalena Knetki-Wróblewska, Dariusz M. Kowalski, Maciej Krzakowski

**Affiliations:** 1 Department of Lung Cancer and Thoracic Tumors, Maria Sklodowska-Curie National Research Institute of Oncology, 02-781 Warsaw, Poland; adam.pluzanski@pib-nio.pl (A.P.); sylwia.tabor@pib-nio.pl (S.T.); kinga.winiarczyk@pib-nio.pl (K.W.); magdalena.knetki-wroblewska@pib-nio.pl (M.K.-W.); dariusz.kowalski@pib-nio.pl (D.M.K.); maciej.krzakowski@pib-nio.pl (M.K.); 2 Department of Pathology, Military Institute of Medicine—National Research Institute, 04-141 Warsaw, Poland; piotr.patolog@gmail.com

**Keywords:** pulmonary benign metastasizing leiomyoma, PBML, postmenopausal, diagnosis, hormone therapy

## Abstract

Pulmonary benign metastasizing leiomyoma (PBML) is a rare condition characterized by the spread of uterine leiomyomas to the lungs, typically observed in premenopausal women with a history of hysterectomy or myomectomy. This report presents a unique case of a postmenopausal woman, aged 65, that emphasizes the clinical, radiological, histologic, and immunohistochemical aspects of the disease. On presentation, the patient suffered from severe pain. On imaging, a sizable lung tumor was found. Histopathological examination and immunoprofiling confirmed PBML. The patient underwent various treatments, including surgery, radiation therapy, and hormonal therapy, illustrating the challenges in managing PBML. A literature review underscores the rarity of PBML and its diverse clinical manifestations. This study provides valuable insights into the complexities of PBML.

## 1. Introduction

Pulmonary benign metastasizing leiomyoma (PBML) is an uncommon and intriguing clinical entity characterized by the metastatic spread of benign uterine leiomyomas to the lungs [1]. It has only been reported in 100 cases worldwide as of 2020 [2]. It occurs predominantly in premenopausal women with a history of uterine leiomyomas, often following hysterectomy or myomectomy [3]. This phenomenon, though rare, has been reported in numerous case studies, prompting researchers to explore the underlying mechanisms and potential therapeutic strategies. In this case report, in addition to adding to the reported cases in the literature, we detail the clinical presentation, imaging studies, features of immunohistochemical staining, as well as the differential diagnostic and therapeutic implications of the precise diagnosis of PBML. Furthermore, we provide a literature review of PBML, aiming to synthesize the current understanding of its pathogenesis, clinical presentation, diagnosis, and management.

## 2. Case Report

**Figure 1:**
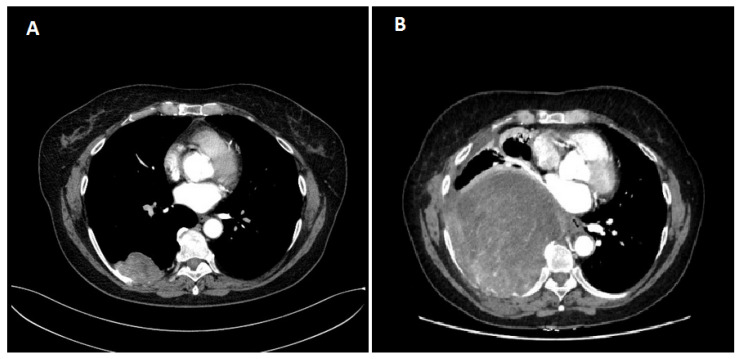
Chest CT scan. (**A**) first; (**B**) last. CT—computed tomography.

**Figure 2:**
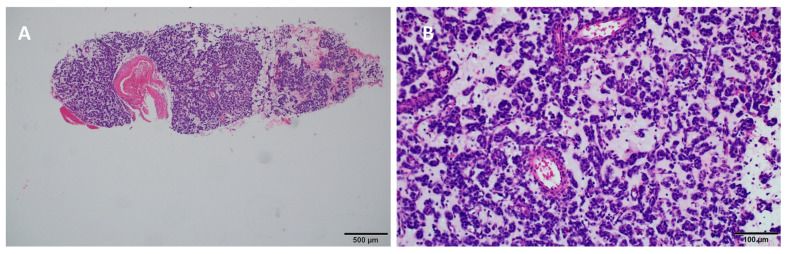
Pathological findings in the right lung through core-needle lung biopsy. Hematoxylin and Eosin stain at (**A**) 40× and (**B**) 200× magnification.

**Figure 3:**
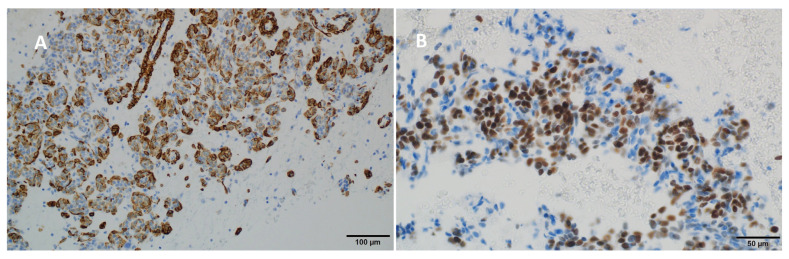
Immunohistochemical staining results showing (**A**) positivity for desmin (200×) and (**B**) positivity for the estrogen receptor (ER) (400×).

**Figure 4:**
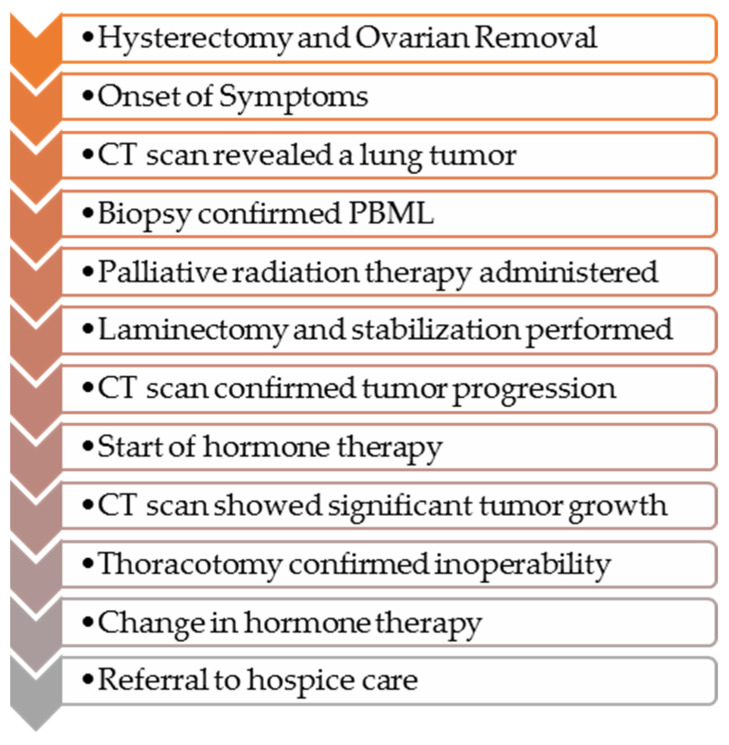
Chronology of events in the presented case.

A 65-year-old white woman began diagnostics due to severe pain that hindered her mobility. The pain was localized to the lumbar spine, right hip, and right thigh. Contrast-enhanced computed tomography (CT) on 20 April 2020, revealed a tumor measuring 58 × 34 mm in the 9/10 segment of the right lung with infiltration and pathological fracture of the eighth right rib at its vertebral end (Figure 1). Additionally, there was destruction and fracture of the L2 vertebral body with a spinal canal stenosis and compression on the dural sac. Laboratory tests showed no clinically significant deviations, and tumor markers were within the normal range. Mammographic and ultrasonographic examinations of the breasts described a benign change in the right breast (BIRADS 2). Magnetic resonance imaging (MRI) of the lumbar spine revealed pathological compressive fracture of the L2 vertebral body with indentation fragments and infiltrative changes into the spinal canal. Neurosurgical consultation did not recommend surgical treatment. A core-needle biopsy of the infiltrating chest lesion on the right side was performed. Histopathology and immunoprofile were atypical for primary lung cancer. The patient had a history of uterine and ovary removal in 2010 due to uterine leiomyomas. The patient’s last menstrual period was at the age of 40. She had no history of hormone replacement therapy. She was a smoker (10 pack-years), but she has abstained from smoking for the past 20 years. Consultation and comparison of specimens on 10 June 2020, confirmed metastasis of benign uterine fibroid to the lungs. The overall microscopic image of the biopsy of the lung lesion corresponds to benign leiomyoma metastasis, with a positive expression of smooth muscle actin (SMA), desmin, estrogen receptor (ER), low Ki-67 index, and absence of necrosis (Figure 2 and Figure 3). Due to symptoms and imaging results, palliative radiation therapy was administered, but a single dose of 800 cGy did not result in significant clinical improvement. The patient underwent pain management consultations, modified analgesic treatment for satisfactory pain control, and a steroid block to the sacral hiatus with good results. Seeking further intervention, the patient consulted another neurosurgical center where a laminectomy of L2 with transpedicular stabilization from Th12-L4 was performed on 24 June 2020. Subsequent imaging on 21 August 2020, confirmed the presence of a tumor in the right lung with infiltration and pathological fracture of the eighth right rib at its vertebral end. The L2 vertebral body showed infiltration, destruction, and fracture with an indentation fragment into the spinal canal, absolute spinal canal stenosis, and compression on the dural sac. Given the risk of paralysis and the absence of viable local treatment options, the medical team initiated a trial of palliative hormone therapy. Over the following years, the patient was under the care of a gynecological clinic, receiving medroxyprogesterone (Provera) from 31 August 2021. A CT scan on 29 November 2021, indicated a significant increase in the right lung tumor to approximately 12 × 12 × 20 cm. Due to the onset of respiratory symptoms, especially increased shortness of breath with minimal exertion, the patient was referred to the Lung Cancer and Thoracic Tumors Department. An MRI performed on 23 February 2022, revealed a massive tumor filling the entire right lung. The solid tumor mass infiltrated the chest wall, the posterior part of the eighth rib, intercostal muscles, and the latissimus dorsi muscle. An exploratory thoracotomy on 7 March 2022, confirmed inoperability due to infiltration of the chest wall, pulmonary hilum, and the inferior and superior vena cava. Tissue material was collected, and microscopic examination confirmed the same diagnosis as in the case of the lung biopsy in 2020 and the uterine tumor in 2010. The patient was re-referred to the gynecological department, and the hormone therapy was changed to gonadotropic-releasing hormone (GnRH) analog and aromatase inhibitor. A follow-up CT scan on 26 October 2022, indicated disease progression (Figure 1). Another change in hormone therapy to megestrol achieved disease stabilization. Because of the poor overall condition, the medical team directed the patient for further symptomatic treatment under hospice care on 19 September 2023, where she died after four months. Figure 4 illustrates the key events in the diagnosis and treatment of the presenting patient.

## 3. Discussion

Benign metastasizing leiomyoma (BML) is a rare condition characterized by the presence of benign-appearing aggregates of smooth muscle cells, which can occur at any site in the body [1,3]. The most common site of metastasis is the lungs, termed Pulmonary Benign Metastasizing Leiomyoma (PBML) [4,5]. The pathogenesis of this disease is still unclear, with several hypotheses proposed. The most widely accepted is that the lung nodules in BML originate from uterine smooth muscle cells that spread after myomectomy or hysterectomy, resulting in surgically induced vascular dissemination [1,6,7,8]. Changes in the lungs are typically identified several years (ranging from 1 month to over 20 years) after hysterectomy or myomectomy [9]. In a study by Fan et al., nearly 70% of patients underwent myomectomy or hysterectomy for uterine leiomyoma before diagnosis of PBML; in two other studies, this percentage was 80% and 100% [3,10,11]. In the presented case, the patient had a similar medical history. However, the case stands out due to the atypical course of this rare disease.

Clinical characteristics of PBML patients described in the literature are highly diverse. It usually affects women in their late reproductive years, and the level of sex hormones is closely related to its clinical course [6,12,13,14,15,16]. The average age at diagnosis is typically 43–47 years [3,6,12,13,14,15,16,17,18,19,20,21,22,23]. Postmenopausal patients, as in the presented case, have also been reported in other studies [3,11,24,25,26,27], although they constitute a small proportion of all PBML patients. Cases of PBML in postmenopausal patients highlight the importance of individual differences in the pathogenesis of this disease. BML tumors frequently express estrogen and progesterone receptors, indicating a potential hormonal dependency for tumor growth. Even in postmenopausal women, low levels of circulating estrogens or other sources of estrogenic activity might stimulate tumor proliferation. In a case report by Krentel and Hucke, disseminated hormone-producing leiomyomatosis was observed following laparoscopic supracervical hysterectomy. They found a correlation between serum estrogen levels and the presence of leiomyomas even after bilateral adnexectomy, suggesting the possibility of autocrine hormone production by the tumors [28]. Hormone replacement therapy (HRT) or residual ovarian function could contribute to the presence of estrogen, thereby influencing the progression of BML [22,29]. The interval between the initial diagnosis of uterine leiomyoma and the identification of pulmonary metastases can span several years to decades. BML can manifest many years post-hysterectomy, suggesting that dormant leiomyoma cells may become active under certain conditions, even long after the cessation of ovarian function [22,30,31]. These cells can remain dormant in the lungs and other tissues, potentially becoming active later due to unknown triggers, which might include minor hormonal changes or other environmental factors [31,32]. Some studies suggest that other growth factors or genetic mutations may contribute to the proliferation of BML cells. Genetic factors, such as specific mutations or chromosomal aberrations, could drive the growth of these cells independently of hormonal influences [22,30]. The growth rate of lung lesions is usually slow [18,33]. In most cases, the diagnosis is incidental, with lung changes detected during routine chest radiological examinations. Some patients may also exhibit nonspecific symptoms, such as cough, wheezing, chest pain, hemoptysis, and recurrent pleural effusion [14,34,35,36,37]. It is worth noting that the main clinical symptoms of BML vary depending on the affected organs. The lungs are the most common site of BML metastasis, while involvement of the spine is extremely rare. In a study by Zong et al., twelve reported cases of spinal involvement were found. Six patients were diagnosed with PBML, six with BML in other locations (bones, abdomen, pelvis, shoulder, thigh, lymph node, kidney, liver, stomach, muscles), two had metastases only to the spine, and two had no other metastases [38]. When lesions are located in the spine, patients may complain of pain and paresthesia due to osteolytic damage and compression of the spinal canal by the tumor, as in our patient.

CT images of PBML are not specific, making diagnosis and differentiation from diseases with a more aggressive course challenging [4,22,24,38]. In imaging studies of most PBML patients, numerous solid nodules (87% of cases) are observed, occurring bilaterally in 70% of cases, with an average number of nodules around six, ranging in size from a few millimeters to several centimeters, and an average nodule size of 1.8 cm. Extensive popcorn-like nodules in both lungs, resembling imaging in tuberculosis, have also been described [36]. Single nodules, as in the presented case description, are observed in only 13% of cases [11,39]. Positron emission tomography in combination with computed tomography (PET-CT) can help differentiate PBML from malignant diseases, as PBML shows significantly lower 18-fluorodeoxyglucose (18-FDG) uptake compared to high uptake in malignant lesions. However, it should be noted that other lung changes without 18-FDG uptake also have a malignant character, such as mucinous component carcinomas (lung, breast, kidney, gastrointestinal tract), neuroendocrine tumors, including carcinoids [5,22,40]. PET-CT was not performed on the presented patient.

Accurate identification of BML nodules can be achieved through proper tissue sampling for histological evaluation. The standard diagnostic procedure involves lung biopsy, complemented by histopathological and immunohistochemical staining. Under the microscope, PBML exhibits histological similarities to uterine smooth muscle leiomyoma, with spindle-shaped smooth muscle cells forming intersecting or scattered bundles, devoid of significant nuclear abnormalities, mitoses, or necrosis [9,10]. Nevertheless, these morphological characteristics lack specificity, as several mesenchymal and epithelial tumors may present similarly [9]. Hence, immunohistochemistry plays a pivotal role in achieving an accurate diagnosis. Typical immunophenotypes of PBML cells include SMA (+), desmin (+), ER (+), and PR (+), with a Ki-67 index usually below 1% [9]. A low mitotic index below 5 mitoses per 10 high-power fields, low Ki67 index, and the absence of cell atypia or necrosis suggests the benign nature of these tumors. On the other hand, a mitotic index greater than 10, distinct cell atypia, and necrosis characterize smooth muscle leiomyosarcomas (LMS). For tumors with features between these extremes, the term “smooth muscle tumor of uncertain malignant potential” is used [11,14,41,42]. Further differentiation is based on the assessment of the presence of estrogen and progesterone receptors. Extrauterine leiomyomas are uniformly ER negative, and only a few (13%) extrauterine leiomyosarcomas show weak and focal ER immunoreaction. In contrast, most PBML are ER positive [14]. In the evaluation of PBML, it is crucial to consider the potential risk of malignant transformation. Differentiation between PBML and low-grade LMS is critical, as the management and prognosis of these conditions differ significantly. PBML follows a benign course, while LMS requires more aggressive treatment because of its malignant potential [18]. Our case of PBML presents a challenge because of its atypical aggressive behavior. Generally, PBML is considered a benign condition with a slow progression; however, instances of rapid growth and severe symptoms have been documented, highlighting the potential for more aggressive courses in some patients. In Table 1, we compare PBML with the typical characteristics of Leiomyosarcoma (LMS).

Treatment options for PBML are diverse and often tailored to the individual patient. Hormonal manipulation remains a cornerstone of treatment due to the hormone-sensitive nature of these tumors. Gonadotropin-releasing hormone (GnRH) analogs, such as leuprolide, have been used effectively to reduce tumor size and stabilize the disease. Aromatase inhibitors, which decrease estrogen production, have also shown promise in managing PBML [14,43,44,45,46,47,48]. Additionally, surgical options such as bilateral oophorectomy can lead to regression of pulmonary lesions by eliminating the primary source of estrogen [49,50,51,52]. In cases where hormonal treatments are insufficient, surgical resection of metastatic lesions may be considered, although this is typically reserved for symptomatic relief or diagnostic confirmation [1,3,9,14,18,48]. Close monitoring may be recommended for young women with PBML, particularly those with asymptomatic nodules [3]. Experimental therapies, including tyrosine kinase inhibitors like imatinib, are being explored due to their potential to target specific molecular pathways involved in tumor growth [6,12,31]. In the presented case, despite the symptoms reported by the patient, the extent of the tumor suggested that surgery might not have been radical. Additionally, the high risk of postoperative complications could have further deteriorated the patient’s quality of life. These factors led to the decision to choose a less invasive, palliative approach.

Literature data show disease control or regression under hormonal therapy in 79% of patients [14]. However, in series involving postmenopausal patients treated with hormonal manipulation, a lack of disease control is often observed [11,14]. In the presented case, the patient experienced PBML growth during hormonal therapy. These cases suggest that there may be biological factors other than hormones controlling PBML, especially in older women. Postmenopausal patients may be less responsive to hormonal therapy [3]. Other researchers suggest a misdiagnosis. PBML with an atypical course may actually be low-grade leiomyosarcomas [14,53].

Each patient should be considered for an individualized treatment strategy based on the size and location of the tumor and hormonal status. In patients with limited nodules, surgical resections are essential. In young asymptomatic women, if nodules are not suitable for resection, conservative treatment is preferable over surgical castration. Disease regression under hormonal therapy can be achieved in 79% of patients. A precise assessment and diagnosis are crucial for prognosis due to the benign course and potential response to hormonal manipulation in PBML and the need for more aggressive management in higher-grade lesions.

The article discusses the diagnostic challenges in differentiating BML from malignant tumors such as leiomyosarcoma. Although biopsy studies did not show malignant features, the possibility of leiomyosarcoma in unsampled areas of the tumor cannot be entirely ruled out. The patient’s clinical history, including a prior hysterectomy and pathological findings of low mitotic activity and no necrosis, led to the diagnosis of BML. We emphasize that both BML and leiomyosarcoma can be difficult to distinguish because of their rarity and overlapping features, which necessitates comprehensive diagnostic evaluations. This highlights the limitations of our study and the importance of further research in this area.

## 4. Conclusions

PBML is an extremely rare clinical entity that presents in varied ways, including asymptomatically. This review highlights the importance of raising awareness among clinicians and pathologists, ensuring accurate diagnosis, and considering appropriate management strategies for affected patients. Case studies will continue to elucidate the pathophysiology and potential targeted therapies to treat this intriguing condition. Table 2 provides a summary of the most recent studies on PBML from the last five years, highlighting advancements in understanding and managing this rare condition. Women should routinely be made fully aware of the risk of BML after hysterectomy.

## Figures and Tables

**Table 1:** Comparison of PBML with Leiomyosarcoma (LMS).

Criterion	PBML	LMS
**Clinical Presentation**	Asymptomatic, rarely respiratory symptoms. In aggressive cases: significant respiratory distress, increased nodule size, systemic symptoms (weight loss, fatigue)	Severe and acute symptoms such as cough, hemoptysis, chest pain, rapid deterioration in respiratory function
**Radiological Features**	Multiple, well-circumscribed nodules. In aggressive cases: diffuse miliary patterns, cavitated nodule	Irregular, invasive masses, single or multiple nodules, often showing aggressive invasion into surrounding tissues
**Histopathological** **Features**	Spindle cells, no atypia, low mitotic index. Aggressive PBML may show higher proliferative activity	Spindle cells with significant atypia, high mitotic index, areas of necrosis
**Immunohistochemistry**	Desmin(+), SMA(+), ER/PR(+)	Desmin(+), SMA(+), sometimes ER/PR(−)
**Hormone Receptor** **Expression**	Often present (ER, PR)	Less commonly present
**Clinical Behavior**	Slow-growing, rarely aggressive. Aggressive cases show rapid growth and severe symptoms	Aggressive, rapid growth
**Treatment**	Hysterectomy, oophorectomy, hormonal therapy. Aggressive cases may require more intensive treatment	Surgery, radiotherapy, chemotherapy
**Prognosis**	Good, dependent on hormonal therapy. Variable in aggressive cases	Variable, often poor

**Table 2:** Recent studies on PBML from the last 5 years (2020–2024).

Authors	Year	Journal	Key Findings
Ventura L et al.	2021	Molecular Biology Reports [54]	Role of miRNA-221 and miRNA-126 in PBML, offering new diagnostic, prognostic, and therapeutic perspectives.
Tamura S et al.	2022	J Clin Med Res [55]	Identified potential biomarkers related to the pathogenesis of uterine benign mesenchymal tumors and PBML.
Zhang G et al.	2024	BMC Medicine [56]	Sirolimus is safe and effective in treating PBML.

## Data Availability

The original contributions presented in the study are included in the article, further inquiries can be directed to the corresponding author.
